# Neutrophils and Malaria

**DOI:** 10.3389/fimmu.2018.03005

**Published:** 2018-12-19

**Authors:** Elizabeth H. Aitken, Agersew Alemu, Stephen J. Rogerson

**Affiliations:** Department of Medicine at Royal Melbourne Hospital, Peter Doherty Institute, University of Melbourne, Melbourne, VIC, Australia

**Keywords:** neutrophil, malaria, *Plasmodium*, immunity, antibody mediated immunity, polymorphonuclear (PMN)

## Abstract

Neutrophils are abundant in the circulation and are one of the immune system's first lines of defense against infection. There has been substantial work carried out investigating the role of neutrophils in malaria and it is clear that during infection neutrophils are activated and are capable of clearing malaria parasites by a number of mechanisms. This review focuses on neutrophil responses to human malarias, summarizing evidence which helps us understand where neutrophils are, what they are doing, how they interact with parasites as well as their potential role in vaccine mediated immunity. We also outline future research priorities for these, the most abundant of leukocytes.

## Introduction

Neutrophils (also known as polymorphonuclear cells) are the most common white blood cell in the body (1). They can clear pathogens by phagocytosis; by producing reactive oxygen species (ROS) and other antimicrobial products; or by formation of neutrophil extracellular traps (NETs) (2). Additionally, they also play a role in the activation and regulation of the immune response, by cytokine and chemokine secretion (3), and possibly antigen presentation (4). Their importance in controlling infection can be highlighted by the increased susceptibility to fungal and bacterial infections seen in individuals suffering from neutrophil deficiencies (5). Malaria is a disease caused by infection with *Plasmodium spp.* parasites. It causes severe morbidity and mortality, and young children and pregnant women are especially susceptible to disease. Malaria is a large public health burden with an estimated 216 million cases of malaria being reported in 2016, resulting in an estimated 445,000 deaths (6). Globally most disease caused by infection with *Plasmodium spp.* is caused by *P. falciparum* (6). Pathology is thought to be due to a combination of the sequestration of infected red blood cells (iRBC) in the microvasculature, endothelial activation, as well as pro-coagulant and importantly pro-inflammatory responses (7). In this review, we assess the literature examining how neutrophils and *Plasmodium spp.* parasites interact, and the mechanisms by which neutrophils can play an active role in parasite clearance.

## Neutrophil Dynamics and Recruitment to Sites of Parasite Sequestration

Changes in peripheral blood neutrophil levels have been described during *Plasmodium spp.* infections. In controlled human malaria infections (CHMI) in non-immune individuals, neutrophil numbers are stable during the asymptomatic liver stage (8). In naturally-infected individuals, patterns of change in peripheral blood neutrophil numbers vary with the cohort studied. Using hematological data from over 3,000 children, Olliaro et al. estimated that peripheral blood neutrophil counts increase about 43% (95% CI 26–35%) during acute uncomplicated malaria, and that the level of increase is positively associated with parasitaemia (9). In semi-immune travelers neutrophil counts were higher in those with severe malaria compared to those with uncomplicated malaria, while in non-immune travelers, though neutrophil counts increased with the presence of infection, neutrophil counts did not vary with disease severity (10). A study in HIV-infected individuals showed no difference in neutrophil numbers when comparing those with and without asymptomatic *P. falciparum* infection (11), whereas pregnant women with *P. falciparum* infection had lower numbers of peripheral blood neutrophils than uninfected women (12). Differences between cohorts are likely due to disease status classification (clinical malaria or asymptomatic parasitemia), immune status and/or age.

Neutrophils are a heterogenous population and this is important because different neutrophil subsets can have varying functional properties, for example CD177+ neutrophils are also positive for Proteinase 3, and IL17+ neutrophils have increased ROS production [reviewed in (13)]. We know that neutrophils from individuals infected with *Plasmodium spp.* behave differently compared to those from non-infected individuals (14–18), and a subset of neutrophils with impaired oxidative burst have been observed in individuals infected with *P. falciparum* (18), suggesting that neutrophil subsets change during the course of infection. In individuals challenged with LPS, inflammation results in the release of a neutrophil subset that suppresses T cell activation (19), whether this occurs during *P. falciparum* infection is unclear but it is one example of why work to identify neutrophil subsets in *Plasmodium spp.* infections would likely yield valuable information into the role of neutrophils in malaria.

Neutrophils are generally the first circulating cells to respond to an invading pathogen. However, how and whether neutrophils are recruited to the sites of iRBC sequestration is still unclear. We know very little regarding neutrophil expression of receptors involved in migration and adhesion. There is no evidence that neutrophil adhesion molecule CD11a changes with infection (18), and expression by neutrophils of other adhesion molecules such as CD18, CD11b, and CD62L is still unstudied. There is more information on the expression of neutrophil receptors on endothelial cells. Expression of receptors on endothelial cells involved in neutrophil adhesion and migration are likely increased with infection. Intercellular adhesion molecule-1 (ICAM-1), vascular cell adhesion molecule-1 (VCAM-1) and the endothelial leukocyte adhesion molecule E-selectin are increased on endothelial cells after exposure to iRBC *in vitro* [reviewed in (20)] and this is supported by observations showing increased levels of soluble E-selectin and soluble ICAM-1 in the blood of *P. falciparum* infected individuals (21). Regarding chemokines involved in neutrophil recruitment, neutrophil chemoattractant protein CXCL8 is increased in peripheral blood of patients with severe malaria [reviewed in (22)] (23) as well as in the cerebral spinal fluid (CSF) of children with cerebral malaria and in the placentas of women with malaria in pregnancy [reviewed in (22)]. In addition, *P. falciparum* antigen can induce the production of neutrophil recruitment chemokines CXCL1 and Interleukin 8 (IL8) production by endothelial cells and the production of Interleukin 8 (IL8) by placental syncytiotrophoblast [reviewed in (22)]. Interestingly, although increased expression of neutrophil chemoattractants occurs, studies of malaria pathology rarely show significant neutrophil infiltration at sites of sequestration.

Low numbers of neutrophils were reported in the brain microvasculature in autopsy samples from children in Malawi (14), and neutrophil numbers were not significantly higher in placentas infected with *P. falciparum* or *P. vivax* compared to non-infected placentas (24, 25). An exception is chronic *P. falciparum* placental malaria, which can be accompanied by massive intervillous inflammation, with increased numbers of CD15+ granulocytes (predominantly neutrophils) (26). When the lungs were studied, one study from Thailand showed no difference in neutrophil levels (measured by the presence of elastase positive cells) between fatal *P. falciparum* malaria with or without pulmonary oedema and controls who died from trauma (27). By contrast, in fatal *P. vivax* infection, interstitial lung infiltrates consisting of CD15+ cells were reported from Brazil. Caveats are that some individuals suffered co-pathologies and there was no non-infected control group for comparison (28).

The “snap shots” of pathogenesis provided by tissue samples, together with neutrophils' short half-life (29), mean that it is hard to exclude neutrophil recruitment to sites of infection, however the data to date suggest recruitment is not always happening; why is this? One possible answer is that *Plasmodium spp.* infection inhibits neutrophil chemotaxis. Neutrophils from individuals with symptomatic *P. falciparum* malaria have reduced chemotaxis compared to non-infected healthy controls (15) and this is restored 7 days after treatment, suggesting the involvement of parasite antigens. Neutrophils from individuals with cerebral malaria have reduced chemotaxis to Interleukin 8 (IL8) and N-formyl-l-methionyl-l-leucyl-phenylalanine (fMLP) (14), and this reduction in chemotaxis may be partly due to increased free heme (14, 18) and/or decreased neutrophil expression of IL8 receptor CXCR2 (as reported in *P. vivax* malaria) (14, 30). Additionally, blood-stage parasite antigens inhibit proinflammatory protein S100-calcium binding protein (S100P) stimulated chemotaxis of the neutrophil cell line HL-60 *in vitro* (31, 32). Whether the reduced chemotaxis observed *ex vivo* explains the lack of neutrophil recruitment to the sites of parasite sequestration is still uncertain and warrants further investigation (20, 21, 33).

## Neutrophils and Parasite Clearance: Phagocytosis, ROS and NETS

### Neutrophil Phagocytosis of *Plasmodium spp.*

**Figure 1:**
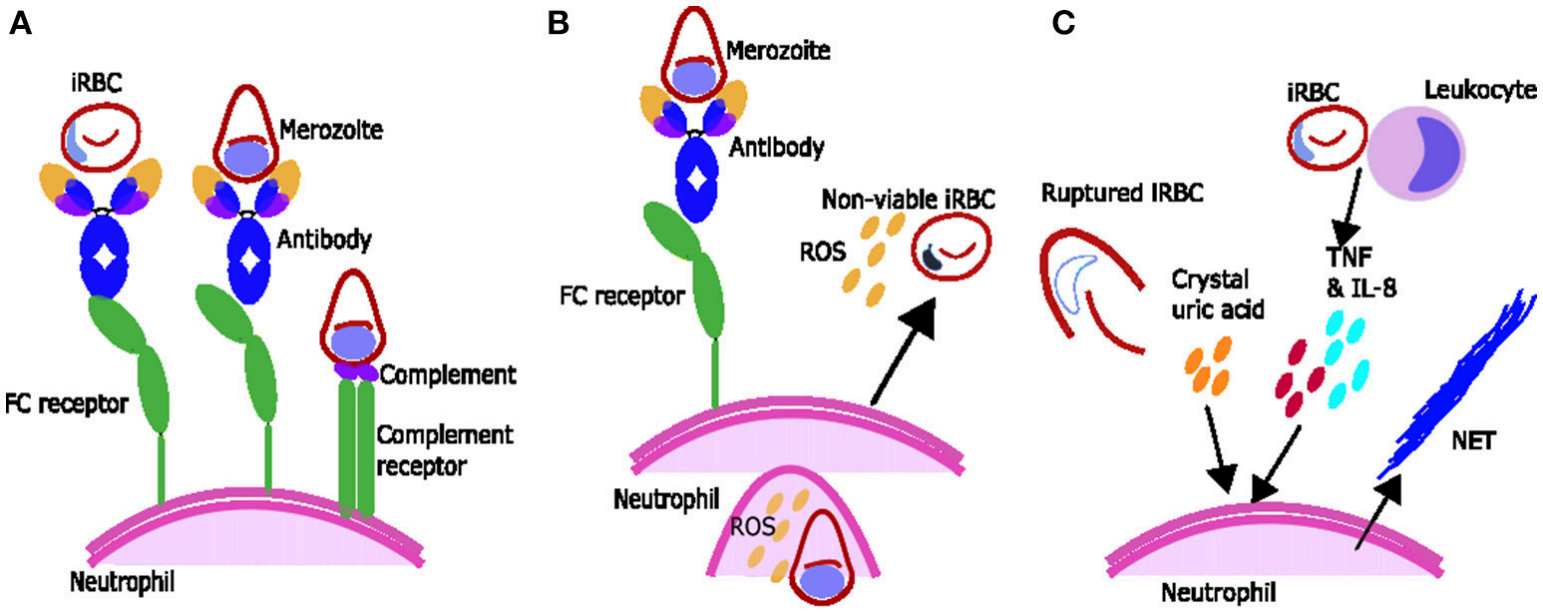
Proposed mechanisms of parasite clearance by neutrophils during *Plasmodium spp.* infection. **(A)** Neutrophils can phagocytose blood stage parasites after opsonization with antibodies and possibly opsonization with complement. **(B)** Neutrophils exposed to merozoite antigen opsonized with antibody produce reactive oxygen species (ROS) which can inhibit intraerythrocytic parasite growth. **(C)** Components associated with *Plasmodium spp.* infection including inflammatory cytokines TNF and IL-8 produced by leukocytes and crystal uric acid released upon infected red blood cell (iRBC) rupture may induce neutrophils to produce neutrophil extracellular traps (NETs).

Phagocytosis is one way that neutrophils play a role in the clearance of malaria parasites. Neutrophils express immunoglobulin (Ig) binding receptors Fcγ receptor I (FcγRI) (after activation), FcγRII and FcγRIII as well as complement receptors complement receptor 1 (CR1) and CR3. Together these can facilitate phagocytic uptake of antigen opsonised with components such as IgG or C3b [reviewed in (34)] (Figure 1A). Neutrophils are known to phagocytose iRBC *in vivo* as observed in blood films from children (35) and in bone marrow aspirates which show phagocytosis of merozoites and occasionally trophozoites by neutrophils and neutrophil metamyelocytes (neutrophil precursors undergoing granulopoiesis) (36).

Neutrophil phagocytosis of merozoites is influenced by several factors. Phagocytosis of merozoites opsonised with non-immune serum has been observed *in vitro* (37) and has possibly been observed *ex vivo* in the blood of ex-service men who had returned from Vietnam infected with *P. falciparum* (38). Neutrophil phagocytosis of merozoites opsonised with non-immune serum is possibly dependent on complement, as heat inactivation decreases phagocytosis, and can be increased if the neutrophils are pre-treated with factors such as tumor necrosis factor (TNF), interferon γ (IFNγ), IL1β or granulocyte-macrophage colony-stimulating factor (GM-CSF) (37). Antibody toward merozoite antigens also promotes phagocytosis, which is much higher if merozoites are opsonised with immune serum compared to non-immune serum (37).

Unlike phagocytosis of merozoites, which may involve complement, phagocytosis of iRBC is largely dependent on the presence of Ig. Neutrophils isolated from malaria infected children can phagocytose schizonts *in vitro* (16), but neutrophils from American service men infected with *P. falciparum* (38) (with limited previous exposure to *P. falciparum* and therefore little Ig toward the iRBC) did not. In addition, sera from individuals living in endemic areas promote phagocytosis of iRBC and this activity is dependent on IgG in the serum (39) and independent of complement (40, 41). It is not known if Ig mediated phagocytosis of parasites by neutrophils is associated with protection from disease.

Intra-erythrocytic gametocytes are not very susceptible to neutrophil phagocytosis (40). However, it is possible that neutrophil phagocytosis of extracellular gametes while in the mosquito gut could play a role in transmission-blocking immunity. Neutrophils phagocytose gametes *in vitro* in conditions similar to those of the mosquito gut when immune sera is present and this phagocytosis is dependent on gametocyte antibodies, especially IgG (40). *In vivo* it has been shown that neutrophils also phagocytose gametes inside mosquito midguts, and that this phagocytosis is enhanced by the presence of immune serum in the blood meal (40). However, although the presence of neutrophils and individual sera in the blood meal decreases infectivity (42), there is no evidence of an association between levels of neutrophil phagocytosis, promoted by serum *in vitro*, and the ability of that serum to affect mosquito infectivity in experimental settings (40).

In rat models, neutrophils phagocytose exoerythrocytic parasites (43), however *ex-vivo* evidence of a role of neutrophil phagocytosis and immunity toward this parasite stage in humans (both *in vitro* and *ex vivo*) is lacking.

Key unanswered questions include the importance of neutrophil phagocytosis in protection from infection or disease and in blocking of transmission. Studies examining associations between antibody induction of neutrophil phagocytosis of different parasite life cycle stages and patient outcomes could help determine the importance of neutrophil phagocytosis in protection from disease.

### Reactive Oxygen Species and *Plasmodium spp.* Infection

Neutrophils can clear pathogens by respiratory burst, the conversion of oxygen to superoxide by nicotinamide adenine dinucleotide phosphate oxidase (NAPDH) oxidase (NOX). This superoxide is converted into hydrogen peroxide and hydroxyl radicals, together referred to as ROS. NOX is located on both the neutrophils plasma and phagosomal membranes and the ROS it produces can diffuse across membranes. This means that ROS are present in the phagosome, intracellular and also extracellular spaces [reviewed in (44)] and can therefore play a role in killing both phagocytosed intracellular as well as extracellular parasites.

ROS from activated neutrophils are capable of inhibiting parasite growth *in vitro*, and studies using various ROS inhibitors or scavengers suggest singlet oxygen, rather than hydrogen peroxide or superoxide, is responsible for this inhibition (45, 46). Growth inhibition by ROS occurs during the intra-erythrocytic development stage of the parasite (45, 46) (Figure 1B), rather than during merozoite invasion of erythrocytes, which is not inhibited by ROS production from stimulated neutrophils *in vitro* (47). *Ex vivo* data suggests that during malaria infection neutrophils are activated to produce ROS and that ROS may have a role in parasite clearance. Neutrophils from children with malaria inhibit parasite growth *in vitro* better than neutrophils from uninfected children or adults (16) and oxygen consumption is higher [indicating activation and production of ROS (48)] in neutrophils from people with symptomatic acute malaria compared to controls (17). In addition, children with faster parasite clearance times have neutrophils which produce more ROS (49).

ROS production by neutrophils is influenced by a number of factors, including the host's genetic background (50). In the presence of antigen neutrophils produce higher amounts of ROS with immune serum compared to non-immune serum (51) or compared to IgG depleted serum (52), supporting a role for IgG-Fc interactions in ROS production (52) (Figure 1B). Regarding complement, one study suggests serum heat inactivation reduces ROS production (51) and another showed that heat inactivation changes the dynamics of ROS production (53). However, the activation of neutrophils to produce ROS by heat-inactivated serum (52) and by purified IgG (52, 53) suggests that the full complement cascade is not necessary.

The ability of Ig to induce ROS has been called antibody dependent respiratory burst (ADRB), and assays measure different components of this process. In solid-phase assays (where the antigen is bound on a plate), ROS are secreted from the neutrophil and this process is largely dependent on FcγRIIa. By contrast, when neutrophils phagocytose whole merozoites the resulting ADRB and associated ROS production occur within the neutrophil are only partially dependent on FcγRIIa (53). Antibodies capable of inducing respiratory burst are acquired with exposure to *Plasmodium spp.* In a small cohort study, sera taken after an immune episode and incubated with merozoites induced more neutrophil ROS production than sera taken during the malaria episode, and sera from adults induced more neutrophil ROS production than sera from young children (54). ADRB toward merozoite antigens was higher in a holo-endemic area compared to a meso-endemic area (52) and increased with age (55), and antibodies capable of inducing respiratory burst have been associated with protection from malaria. Individuals whose serum induced a high ADRB toward merozoites were less likely to experience clinical malaria when compared to those whose serum induced a low ADRB (52, 55), and a combined measure of ability of an individual's serum sample to induce both ADRB and growth inhibition has been associated with protection from severe malaria (56). Supporting a role for ADRB in protection from disease, polymorphisms of FcγRIIIb [a neutrophil receptor that is involved in IgG dependent ROS production (57)] that improve neutrophil Fc-Ig binding are associated with protection from febrile malaria (58, 59).

Antibodies opsonising merozoites or merozoite antigens can induce ROS production by neutrophils, whereas there is no evidence that antibodies opsonising iRBCs result in a major ROS response (17, 51, 52, 54, 60). The reasons for this difference are unclear as IgG are known to recognize the surface of iRBC (61). The merozoite antigens which are targets for ADRB include *Plasmodium falciparum* merozoite surface protein-5 (PfMSP5), MSP1-19 and MSP1, as indicated by correlations between IgG antibodies to these antigens and ADRB (55, 62, 63), and by antigen/antibody depletion assays with MSP1 or its C terminal domain MSP1-19 (62, 63). See Figure 1B for diagram for ROS production.

Antibodies which induce ROS may be similar to those which promote merozoite phagocytosis, as the functions are correlated in neutrophils responding to opsonised merozoites (54). Antibody subclass may be important: mouse-human chimeric IgG toward MSP1-19 is sufficient to induce NADPH-mediated oxidative burst (and degranulation) from neutrophils, but mouse-human chimeric IgG3 toward the same antigen is not (64). Multiple antibody isotypes may be involved ROS production. Recombinant human IgA toward merozoite antigen is a potent inducer of ROS (65), but IgA's importance in ROS production in malaria is unclear as IgG depletion from serum appears to be sufficient to eliminate most ADRB toward merozoite antigen (52). Further research is needed to identify the characteristics of antibody responses to malaria antigens that effectively elicit neutrophil ADRB.

### Evidence for NETS in Malaria

**Figure 2:**
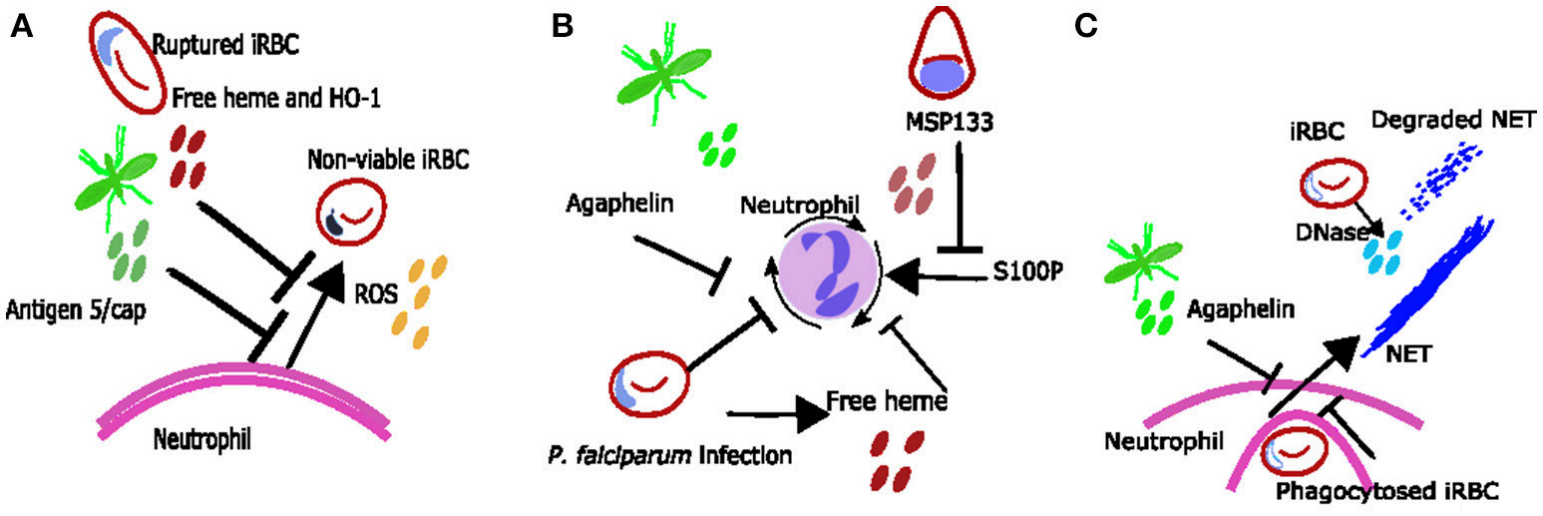
Proposed mechanisms neutrophil inhibition during *Plasmodium spp.* infection. **(A)** Neutrophil ROS may be inhibited due to excess free heme and HO-1 or due to mosquito salivary protein antigen 5/cap. **(B)** reduced neutrophil chemotaxis may be due to exposure to free heme, merozoite protein MSP133 or mosquito salivary protein agaphelin. **(C)** It is possible that phagocytosis of bulky parasite antigens inhibits neutrophils from producing NETs during *Plasmodium spp.* infections and that any NETs produced are degraded by parasite DNase.

Neutrophil extracellular trap (NET) formation has evolved as an important innate strategy for killing extracellular pathogens, and occurs when activated neutrophils degranulate and release neutrophil antimicrobial factors into the extracellular environment. NETs are mesh-like extracellular structures made up of decondensed chromatin and histones decorated with different antimicrobial granular proteins that can capture, neutralize and kill a diversity of microbes (66). There are several factors which might induce NET formation during *Plasmodium* infections (Figure 1C). Crystal uric acid [a potent inducer of NETosis (67)] and its precursor hypoxanthine are released upon iRBC rupture [reviewed in (68)]. In addition, cytokines such as TNF and IL8 which are increased during *Plasmodium* infections [reviewed in (69)], and H_2_O_2_ secreted by immune cells stimulated by *Plasmodium spp.* antigen [reviewed in (70)] have been shown to induce NETosis *in vitro* [reviewed in (67)] (Figure 1C). NET-like structures which stain positive for DNA with 4′,6-Diamidino-2-Phenylindole, Dihydrochloride (DAPI) have been found in peripheral blood of children with uncomplicated *P. falciparum* infections (71), however it is unclear if these structures were produced in response to *Plasmodium* antigen (as opposed to another stimulus) and/or if they are derived from neutrophils (as opposed to monocytes) (72). There is some qualitative evidence that neutrophils can produce NETs in response to *P. falciparum* antigen *in vitro* (73) however, quantitative evidence that neutrophils produce NETs in response to *P. falciparum* and evidence that NETs are present in tissues and contribute to pathology of *Plasmodium* infection in humans is still lacking. When brain tissue sections from 4 children with fatal cerebral malaria (CM) and associated retinopathy and 5 with CM without retinopathy (indicating an alternative diagnosis) were stained with antibodies toward NET markers neutrophil elastase and citrillinated histones, no NETs were seen (14). There are no published studies looking for NETS in other human tissues. Possible explanations for the lack of evidence of NETs in *ex-vivo* brain tissue include neutrophils making NETs that the parasites break down. *P. falciparum* asexual blood stage parasites expresses a DNase virulence factor which may be able to break down NETs, as they are made of DNA (74) (Figure 1C). Alternatively, it may be that NETs are simply not being formed. If neutrophils phagocytose bulky antigens via opsonisation and/or phosphatidylserine (PS) exposure sometimes they cannot make NETs [reviewed in (75)] (Figure 1C) and we know during malaria infection neutrophils do phagocytose bulky antigens (such as merozoites and iRBC) by these mechanisms (Figure 2) (37, 39, 76). Further, research is needed to clarify whether NETs do play an active role in *Plasmodium spp.* infections.

### Neutrophils, Severe Malaria and Parasite Adhesion

As well as playing a role in parasite clearance, neutrophils may contribute to the pathology of severe malaria syndromes, but there is limited supporting data from human studies on this topic. In CM, neutrophil proteins in plasma [including neutrophil primary granule proteins, neutrophil elastase, myeloperoxidase and proteinase 3 (PRTN3)] are associated with disease (14) and it has been hypothesized that neutrophil products contribute to CM pathology, with elastase damaging the endothelium (77, 78), and inflammatory factors such as TNF [reviewed in (79)] and ROS [reviewed in (80)] increasing expression of the iRBC adhesion receptor, ICAM-1 (81) on endothelial cells to promote parasite adhesion. Also, PRTN3 can cleave endothelial protein C receptor (EPCR) from endothelial cells and contribute to the procoagulant state observed in severe malaria [reviewed in (14)]. On the other hand neutrophil products may also reduce parasite adhesion, for example by PRTN cleavage of EPCR (82) as it is a receptor for parasites associated with severe malaria (83). Also, neutrophil elastase, released by activated neutrophils, can cleave iRBC antigens involved in *P. falciparum* iRBC cytoadherence to C32 melanoma cells (84).

It has also been suggested that neutrophils play a role in the pathology of the liver during *Plasmodium spp.* infections (85). Neutrophil activation by type I Interferon is associated with increased serum levels of transaminases in *P. vivax* malaria and together with murine data showing type I IFN modulates neutrophil migration to the liver in mice, suggests type I IFN are responsible for neutrophil mediated liver pathology in malaria (85). Human evidence showing that neutrophils are present in the liver during infection and could cause damage is lacking. Further studies using human samples to identify the role of neutrophils in severe malaria are needed.

### Factors Inhibiting Neutrophil Responses

Although neutrophils can clear parasites in a variety of ways, several mosquito and parasite antigens can modify neutrophil responses to the parasite's advantage (Figure 2). For example, mosquito salivary proteins can alter neutrophil function. Secretion of the protein agaphelin in the mosquito's salivary glands is increased upon infection with *P. falciparum*, and agaphelin can inhibit neutrophil elastase activity, neutrophil chemotaxis and NET formation in response to phorbol myristate acetate (PMA) (31) (Figure 2). Other salivary proteins that may also alter neutrophil function include the antigen-5 salivary proteins, which scavenge superoxide and inhibit neutrophil ROS (86) (Figure 2A).

Parasite antigens which can inhibit neutrophil function include histamine-releasing factor (an ortholog of mammalian histamine-releasing factor), which was shown to inhibit neutrophil IL6 production in the liver in a murine malaria model, and subsequently promoted liver stage parasite development (87). Also, *P. falciparum* protein MSP1-19 can block neutrophil responses to proinflammatory protein S100P, inhibiting neutrophil chemotaxis *in vitro* (32) (Figure 2B). There is clearly an interesting dynamic between neutrophils and *Plasmodium*, with each trying to take advantage and control the other. Identification and understanding of neutrophil parasite interactions may result in the identification of novel therapeutic targets and warrants further investigation.

Parasite-neutrophil interactions may also result in susceptibility to other diseases. Malaria infection (especially with severe anemia) has been associated with susceptibility to non-typhoidal salmonella (NTS) bacteraemia (88, 89) and this is thought to occur due to impaired neutrophil function. *In vitro* observations suggest that neutrophil phagocytosis of parasite products results in them being less capable of phagocytosing bacteria (47). *Ex vivo* observations show reduced ability for neutrophils to generate ROS during malaria (18), and this reduced function was associated with increased haemolysis and heme oxygenase-1 (HO-1) expression in infected individuals (18) (Figure 2A); similar findings have been reported in asymptomatic infection (90). Mouse data suggests that haemolysis during infection induces HO-1 and results in impaired maturation of neutrophils (91). In addition mouse data shows that IL10 [which is also associated with infection in children (90)] inhibits neutrophil migration resulting in altered clinical presentation of NTS (92).

### Pigmented Neutrophils as a Marker of Disease Severity

Neutrophils with malaria pigment can be seen in the peripheral blood during *Plasmodium* infection. In children, the percentage of neutrophils with pigment in the peripheral blood increases with disease severity (93, 94) and is positively correlated with parasitaemia (93, 95). The percentage of neutrophils with pigment has also been positively associated with mortality due to severe malaria in adults and children (94, 96), and in parasitaemic pregnant women high numbers of pigmented neutrophils in the peripheral blood during gestation were associated with lower birth weights at delivery (97). The predictive value of pigmented neutrophils (94, 96, 97) suggest that they could indicate sequestered biomass, and because pigment-containing neutrophils are cleared from the circulation after about 72 h (29) it is likely they are a marker of recent pigment phagocytosis. Pigment containing neutrophils have also been associated with disease severity in *P. knowlesi* malaria (98).

### Neutrophils and Antimalarials

A number of *in vitro* studies have investigated whether neutrophil activity may be affected by antimalarial drugs. At non-physiological levels chloroquine, quinine, proguanil, and mefloquine all inhibit neutrophil oxidative burst (99) however at lower physiological plasma levels they, as well as sulphadoxine, cycloguanil, pyrimethamine and tetracycline, have no depressive effects on oxidative burst nor markers of oxidative metabolism (99). Likewise at high concentrations, chloroquine decreases the phagocytic activity of neutrophils and quinine, chloroquine and quinacrine inhibit neutrophil chemotaxis but at physiological concentrations they do not (100, 101). There is however some evidence that neutrophil chemotaxis and neutrophil iodination (a measure associated with phagocytosis and oxidative burst) are inhibited at physiologically relevant concentrations by pyrimethamine and by mefloquine or pyrimethamine respectively, (101). Mefloquine inhibits oxidative burst by interacting with cellular phospholipid-dependent protein kinase C (102). Amodiaquine and pyronaridine have been shown to cause neutrophil glutathione depletion in *in vitro* systems where pyronaridine is oxidized to a quinonimine metabolite, raising concerns they could be cytotoxic to neutrophils at physiological concentrations (103). However, *in vivo* studies in rats did not find any quinoneimine metabolites after receipt of pyronaridine nor is there is clinical evidence of significant toxicity associated with pyronaridine use in humans [reviewed in (104)].

As well as effects on neutrophil function, studies have raised the possibility that neutrophil numbers may be affected by antimalarials (105–107). In an analysis of data from 7 randomized trials across 13 sites in 9 countries comparing artesunate-amodiaquine to single and combination treatments (including amodiaquine mono-therapy, artesunate mono-therapy, artemether-lumefantrine, artesunate and sulphadoxine-pyrimethamine, and dihydroartemisinin) the treatment-emergent adverse event incidence of neutropenia was 11% (107). However, when neutrophil counts were compared between treatment groups there was no apparent differences (107). Interestingly, in the case of artesunate, an effect on neutrophil numbers may be dose dependent. In a clinical study conducted among Cambodian patients with uncomplicated malaria, those who received 6 mg/kg/day had lower neutrophil counts than those who received 2 or 4 mg/kg/day, also 5 of 26 patients who received 6 mg/kg artesunate developed neutropenia <1000/mm^3^, while only 1 of 38 patients receiving 2 or 4 mg/kg/day did (105).

### A Brief Overview of Neutrophils in Animal Models of Malaria

Murine models have been used to study the role of neutrophils in malaria complications including lung injury, CM and liver injury. Accumulation of neutrophils in the lungs has been associated with lung injury in multiple murine malaria models (73, 108–114) and lung injury is associated with increased TNF (108, 109), parasite sequestration in the pulmonary vasculature (113), the presence of NETs (73), neutrophil adhesion to endothelial walls, and with increased vascular permeability (110, 111) but not ICAM-1 expression (114).

In murine models of CM, neutrophils have been shown to express cytokines IL2, IL12p40, IL18, IFNγ, and TNF as well as chemoattractive-chemokines monokine-induced by gamma (MIG), macrophage inflammatory protein-1α (MIP1α) and IFNγ induced- protein 10 (IP10) (115) suggesting a role for neutrophils in cytokine and chemokine secretion. In murine CM neutrophils are detected in the microvasculature (116), and neutrophil depletion results in decreased monocyte sequestration and microhaemorrhages in the brain and prevents development of CM (117). Other neutrophil factors associated with CM include neutrophil secretion of CXCL10 which may contribute to high parasitemia and disease (118), and neutrophil expression of FcεRI (a high affinity IgE receptor) which may result in the production of proinflammatory cytokines (119).

Studies have identified a possible role for neutrophils in liver damage in murine models with neutrophil dependent liver damage being associated with free heme, NFkB activation and neutrophil infiltration (120). Neutrophil infiltration and subsequent liver damage may also be dependent on type I IFN signaling (85).

Murine models can play a valuable role in dissecting the role of neutrophils in disease, but whether the roles of neutrophils in humans and murine malaria are similar is still unclear and more work needs to be done to validate findings in humans.

### A Role for Neutrophils in Vaccine Mediated Immunity

Neutrophil-antibody interactions can play an active role in parasite clearance and should be considered when evaluating vaccine mediated immunity. ADRB toward merozoite antigens is associated with protection (52, 56), but whether other antigens such as those on the asexual iRBC, gametocytes or sporozoites can trigger ADRB, or whether such responses could be associated with protection, is unknown. Likewise, although it is clear that neutrophil phagocytosis of parasite antigen, merozoites, iRBC and possibly gametocytes occurs *in vivo* (35, 36, 40, 121), it is unknown whether antibodies promoting neutrophil phagocytosis are protective. Studies examining antibody mediated neutrophil functions to a variety of antigens and their associations with protection will help elucidate the role neutrophils have in antibody mediated immunity and their potential in vaccine mediated immunity.

A few studies have used animal models to investigate the role of antibody-dependent neutrophil responses in the context of protection from disease and also vaccination. In one study, murine IgG1 toward MSP1-19 was effective at inducing human neutrophil ADRB and degranulation, but these same antibodies did not protect against *P. berghei* expressing MSP1 *in vivo* (122). In another study, serum from previously challenged mice and both murine IgG1 and IgG2 could elicit ADRB from murine neutrophils in response to murine antigens *in vitro*, however vaccination of mice with MSP1-42 resulted in antibodies which did not elicit neutrophil ADRB toward merozoites and ADRB did not contribute to vaccine mediated protection (123). On the other hand, when non-human primates were immunized with MSP-1, antibodies were produced which opsonised merozoites and elicited ADRB by neutrophils (63), suggesting that vaccination can result in generation of antibodies which activate neutrophils.

As well as their role in parasite clearance by antibody dependent mechanisms, neutrophils may also play a role in immune responses to vaccines. There is growing evidence that neutrophils have the capacity to present antigen to T-cells. Whilst they may not be as effective at antigen presentation as typical antigen presenting cells their overall impact may be significant due to their sheer numbers [reviewed in (4)]. At least in the case of irradiated sporozoites, data from murine models suggests that neutrophils are available to take up the vaccine as intradermal injection of both irradiated and wild type sporozoites result in recruitment of neutrophils (and inflammatory monocytes) to the injection site, however there was no evidence that neutrophil depletion in this model affected the establishment of a protective immune response (124). The occurrence and significance of antigen presentation by neutrophils during both natural malaria infections and vaccination is yet to be investigated.

## Future Directions

Neutrophils in malaria remains understudied, undertaking the future research priorities listed below will go a long way in helping us to understand the role of neutrophils during *Plasmodium spp.* infections.

1. Identify the presence (or absence) of neutrophils at sites of parasite sequestration using *ex vivo* samples from humans.
2. Identify neutrophil subsets during infection in humans using *ex vivo* samples from humans.
3. Identify and quantify neutrophils products (including NETs) at sites of parasite sequestration and in the periphery using *ex vivo* samples from humans.
4. Investigate possible roles of neutrophils in asymptomatic *Plasmodium spp.* infection, clinical malaria and severe malaria by comparing neutrophil counts and indicators of neutrophil activation and/or inhibition between different clinical groups.
5. Using *in vitro* models investigate the role of parasite products (such as DNAse) on neutrophil function.
6. Measure antibody mediated functions of neutrophils (such as ADRB & phagocytosis) and investigate their associations with protection from disease.
7. Using *in vitro* models clarify the role of complement in neutrophil parasite interactions.
8. Identify whether neutrophils should they be considered in evaluation of antibody mediated immunity provided by vaccines.
9. Investigate the role of neutrophils in antigen presentation in the context of both natural infection and vaccination.

## Conclusion

The role of neutrophils in protection and disease during *Plasmodium spp.* infections has been little studied, and important questions remain. Further research with a focus on neutrophil responses toward the parasite and how neutrophils play a role in parasite clearance will likely aid in the development and evaluation of vaccines for malaria.

## Author Contributions

All authors listed have made a substantial, direct and intellectual contribution to the work, and approved it for publication.

### Conflict of Interest Statement

The authors declare that the research was conducted in the absence of any commercial or financial relationships that could be construed as a potential conflict of interest.
